# Supplementary material for: Cost-effectiveness of routine adolescent vaccination with an M72/AS01E-like tuberculosis vaccine in South Africa and India
Source: Nat Commun. 2022 Feb 1;13:602. doi: 10.1038/s41467-022-28234-7 (PMC8807591; doi:10.1038/s41467-022-28234-7)
Supplement: Supplementary file 1 — Supplementary Information [file 41467_2022_28234_MOESM1_ESM.pdf]

Cost-effectiveness of routine vaccination with the M72/AS01$_E$ tuberculosis vaccine in South Africa and India

Online Supplementary Materials

Authors: Rebecca C Harris+, Matthew Quaife*, Chathika Weerasuriya, Gabriela B Gomez, Tom Sumner, Fiammetta Bozzani, Richard G White*

*Joint first-authors, +Corresponding author

Table of Contents

SUPPLEMENTARY METHODS **2**

1. MODEL STRUCTURE, PARAMETERISATION AND CALIBRATION **2**

2. COST MODELLING AND COST-EFFECTIVENESS ANALYSES **5**

CALCULATION OF DISABILITY-ADJUSTED LIFE YEARS (DALYS) 5

TB-RELATED COST MODEL 5

SUPPLEMENTARY RESULTS **8**

SUPPLEMENTARY REFERENCE LIST **21**

Supplementary Methods

1. Model structure, parameterisation and calibration

Model structure, calibration, and parameterisation has been described in detail elsewhere(1), so we present a brief overview here. We developed age- and vaccination status-stratified compartmental deterministic models of *M.tb* transmission in R. The model was calibrated to age-stratified epidemiological TB data for India (13 calibration targets) and age- and HIV status-stratified data for South Africa (16 calibration targets), as in Table S1. UN population division 2017 revision demographic data were used to parameterise population demographics for both countries. Model inputs to reproduce the age-stratified UN demographic estimates and predictions were the UN population division birth rates per 1000 population and probability of death for an individual of a given age group in a given time period for 1950-2050. A manually adjusted calibration factor for background mortality was employed to reflect migration. Country-specific epidemiological factors were incorporated, including private and public sector care in India, and the HIV epidemic in South Africa. HIV epidemiology was parameterised using age-and year-specific HIV-incidence and AIDS-related mortality for South Africa from Spectrum data and projections, and ART was parameterised using data from Spectrum histoically, scaled up to 90% coverage by 2022 to meet the 90:90:90 targets, and held at 90% coverage beyond 2022. Age-wise heterogeneous social mixing patterns parameterised social contacts in both settings. BCG coverage at birth was assumed to remain at current levels, as were other TB control measures. Multistage model calibration was used to incorporate uncertainty in natural history parameters, fitting was initiated with random sampling and, where required, was followed by approximate Bayesian computation Markov chain Monte Carlo. Epidemiological outcomes were estimated from 1000 parameter sets from fitted models, summarised using median, and range (credible interval).

In this study, we model the population-level impact of three key vaccine characteristics: age of routine vaccination (10-, 15- and 18-year olds), coverage of the vaccination campaign (80% among 10- and 15- year olds, 50% among 18 year-olds), and vaccination efficacy by host infection status (post-infection efficacy only, and pre-and post-infection). Vaccine implementation was modelled as annual routine vaccination of single- year age groups during 2025-2050, vaccine efficacy was modelled to be degree (“leaky”), providing 15-years duration of protection, waning instantly at the end of the protection. Vaccine was assumed safe and effective in HIV-positive populations. The main epidemiological outcome was percentage incidence rate reduction (IRR, %) in 2050, comparing each vaccination scenario to the no vaccine baseline scenario. Figure S1 demonstrates the how the vaccine efficacy types and by host infection status impact the natural history of TB.

**Calibration Target**	**Year**	**India**	**South Africa**
**Prevalence**	2015	All age	All age
**Incidence rate**	2000	All age, <15, ≥15	All age, HIV stratified
	2015 or 2016	All age, <15, ≥15	All age, <15, ≥15, HIV stratified
**Mortality rate**	2000	-	All age, HIV stratified
	2015	All age	All age, HIV stratified
**Notification rate (Treatment initiation in India)**	2000 or 2007	All age, ≥15	All age, HIV stratified
	2010 or 2015	All age, <15, ≥15	All age, <15, ≥15, HIV stratified
**TOTAL NUMBER OF TARGETS**	-	**13**	**16**

Supplementary Table 1: Calibration targets for South Africa and India

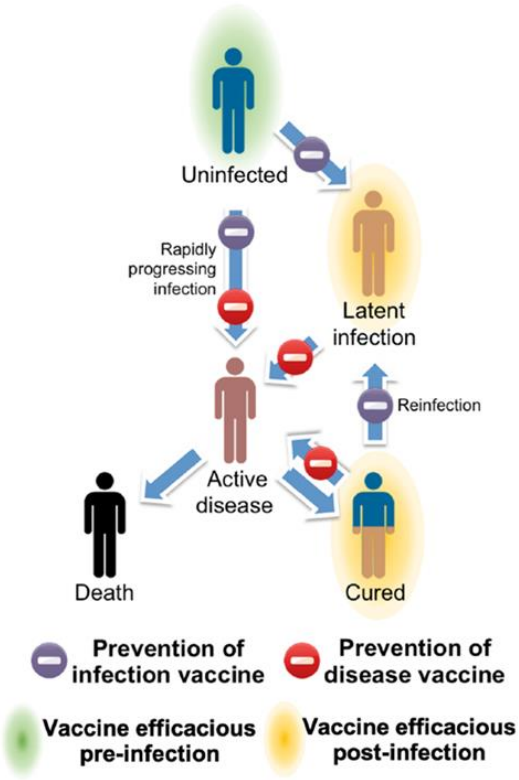

Supplementary Figure 1. Vaccine characteristics. Effect on the natural history of TB disease by vaccines that help prevent infection (purple) or disease (red) and in populations pre-infection (green) or post-infection (yellow). In this study we modelled vaccines efficacious against disease (red), and efficacious either post-infection (yellow) or pre- and post infection (yellow and green).

2. Cost modelling and cost-effectiveness analyses

Calculation of disability-adjusted life years (DALYs)

We calculated the difference in total disability-adjusted life years (DALYs) incurred by baseline and vaccination scenarios. We used the disability weight for TB disease (0.333) from the Global Burden of Disease 2019 study (2), and country- and age-specific life expectancy estimates from the United Nations Development Programme (3).

TB-related cost model

We build off earlier cost modelling work in India (4) and South Africa (5) to estimate costs from a health service perspective using an ingredient approach (Table S2). We obtained unit costs for drug susceptible (DS) and drug resistant (DR) TB treatment, diagnostic costs and tested:diagnosed ratio(6-9). In South Africa we include HIV treatment costs due to the close relationship between HIV and TB epidemics(10). In both settings we include the cost of nutritional support delivered alongside treatment. As elsewhere (4), In India we explicitly include a financial incentive paid to the private sector to encourage case notification. In addition to base case analyses, we take a societal perspective and incorporate patient costs from accessing TB care(11, 12). Uncertainty in cost estimates is characterised through a gamma distribution in a probabilistic sensitivity analysis.

There was considerable uncertainty in the cost of delivering a vaccine, including the price of vaccine compounds and programmatic delivery among adolescents. Based on expert opinion from funders, in both countries we assume a $5 per-recipient vaccination cost, which incorporates the cost of the vaccine and delivery. Meta-analysis evidence suggests that delivery costs (not including the vaccine itself) of childhood vaccination across low- and middle-income countries (LMICs) was $1.87 (95% uncertainty interval $0.64–4.38)(13). One vaccine has been recently widely introduced through routine vaccination among adolescents in LMICs to prevent human papillomavirus. The three-dose Gardasil HPV vaccine cost around $10 per dose per person in South Africa(14), and $2/person vaccinated in India(15). To parameterise uncertainty in this estimate in the probabilistic sensitivity analysis we take the average difference between the two countries.

Unit cost	Central estimate (USD$)	Lower-upper bound (USD$)	Source	Notes
South Africa				
DS TB treatment per person treated*	165	93-287	(7)	
DR TB treatment cost per person treated	6163	2543-9784	(6, 7)	Uncertainty assumed proportionate to that of DS-TB treatment costs. Costs of DR treatment split across 2 years in ratio 2:1 as 18 month course.
Patient nutritional support	7.46	6-8.9	(16)	Bounds assumed +-20%
Ratio tested:diagnosed	1.16		(5)	
Cost per person tested	24	24-25	(17)	
ART costs (children, per year)	284	227-340	(10)	Bounds assumed +-20%
ART costs (adults, per year)	250	200-300	(10)	Bounds assumed +-20%
Patient cost per TB disease episode	324	81-1296	(11)	Uncertainty range based on uncertainty in India 2020
Vaccination price and delivery cost	5	1-9	Funder input	Variation based on HPV vaccination implementation cost (14)
India				
DS TB treatment per person treated	317	254-374	(6, 8, 9)	
DR TB treatment cost per person	3891	3382-4401	(6, 8, 9)	Costs of DR treatment split across 2 years in ratio 2:1 as 18 month

treated				course
Private sector incentive - per patient treated in private sector	3.73	Not varied	(16)	
Patient nutritional support	7	Not varied	(16)	
Cost per person diagnosed	56	45-67	(18)	
Patient cost per TB disease episode	324	81-1296	(12)	Median of estimates reported
Vaccination price and delivery cost	5	1-9	Funder input	Variation based on HPV vaccination implementation cost (15)

Table S2. Unit cost parameters for South Africa and India. Gamma distribution used to simulate uncertainty in costs in probabilistic sensitivity analysis.

Supplementary Results

Figures S2 to S5 summarise the baseline model fit to all calibration targets, without vaccine introduction. Figure S6 shows estimated annual incidence number in baseline and vaccination scenarios. Figures S7 and S8 shows cost-effectiveness planes and incremental cost-effectiveness acceptability curves when patient costs are included.

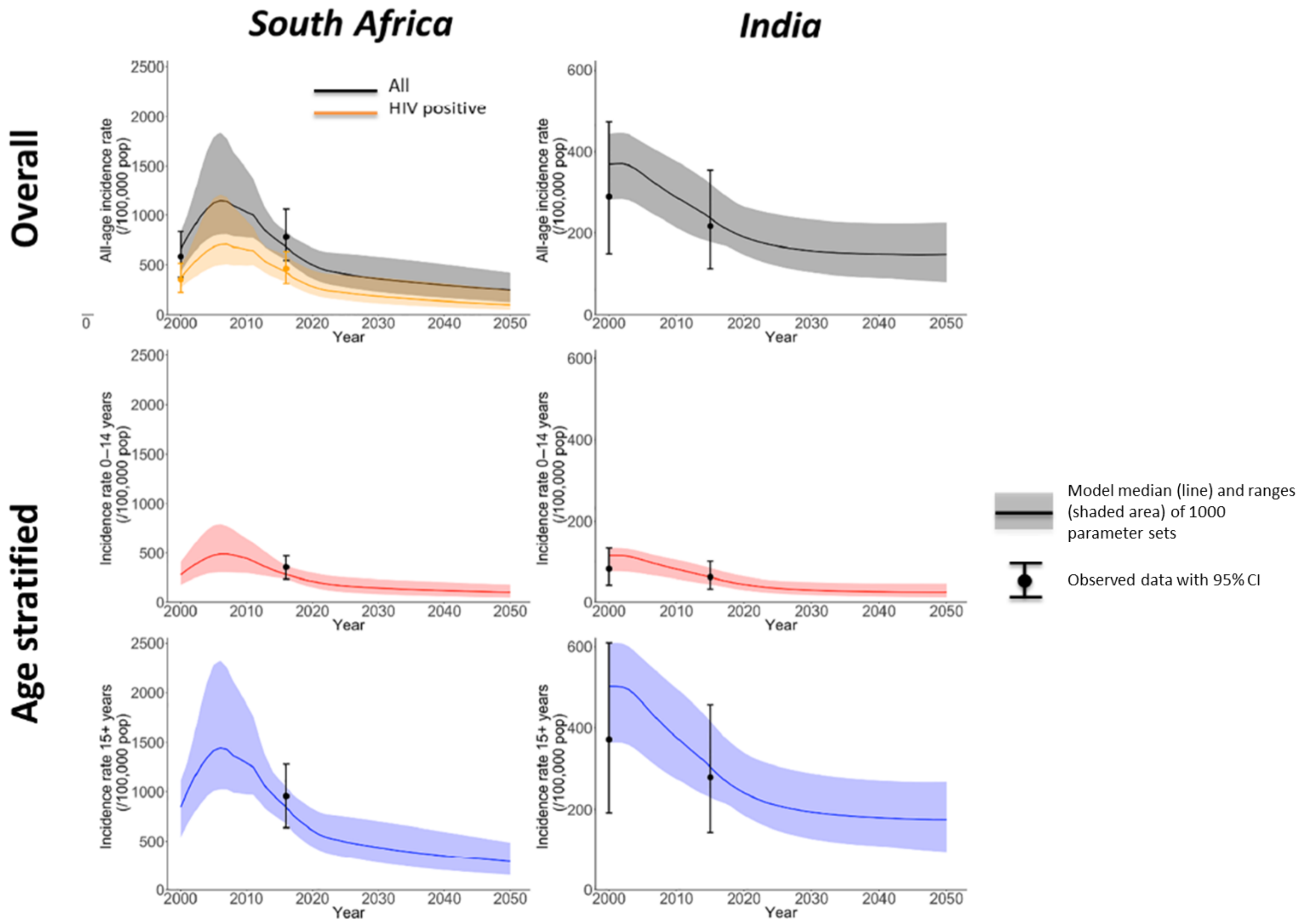

Supplementary Figure 2: Incidence rate projection from calibrated models. Incidence rates shown over time for South Africa and India. Median (line) and ranges (shaded area) summarise 1000 calibrated parameter sets for each country. NB. Axes differ to aid visualisation.

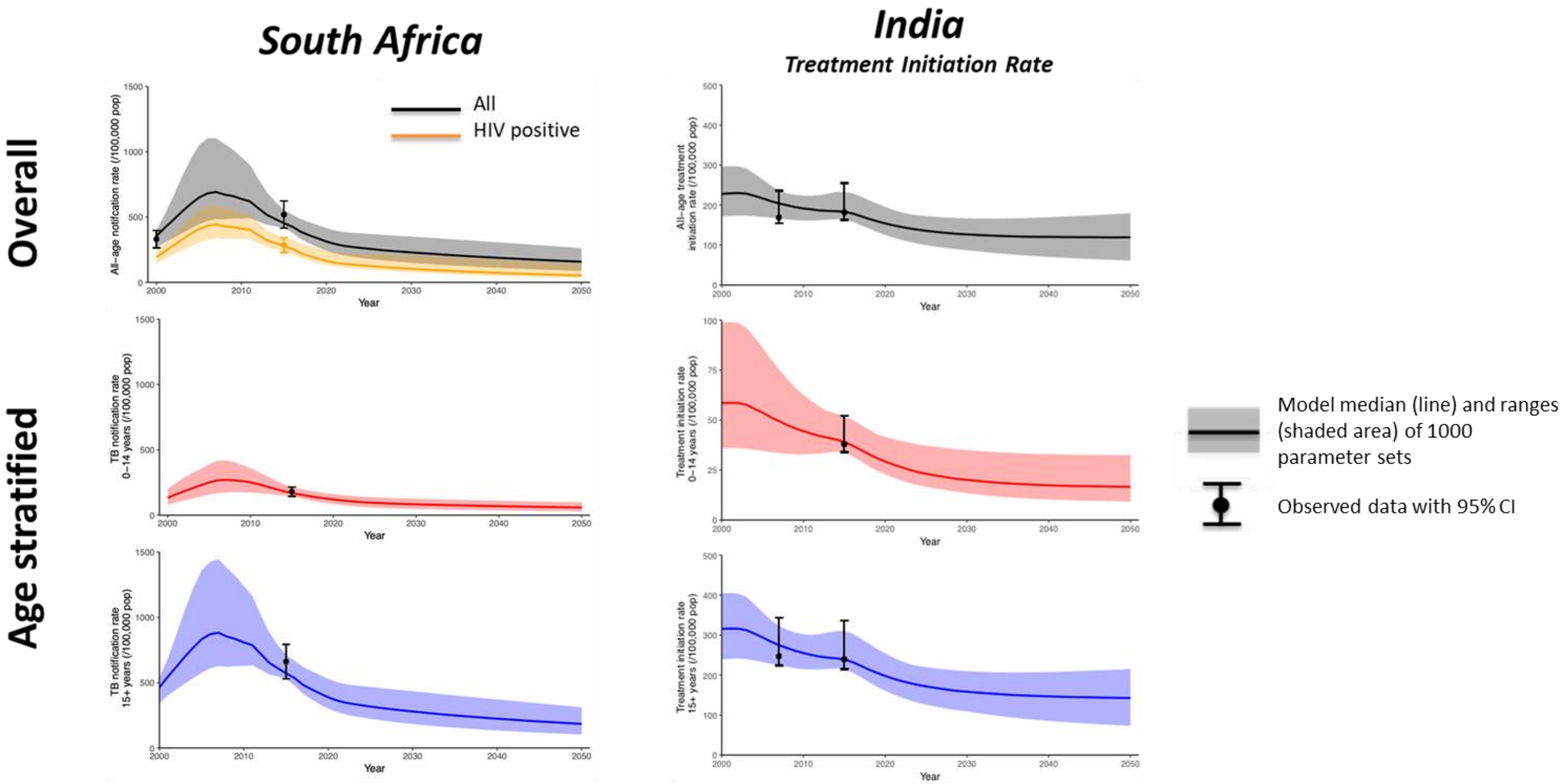

Supplementary Figure 3: TB notification rate or treatment initiation rate projection from calibrated models. Notification or treatment initiation rates shown over time for South Africa and India (respectively). Median (line) and ranges (shaded area) summarise 1000 calibrated parameter sets for each country. NB. Axes differ to aid visualisation.

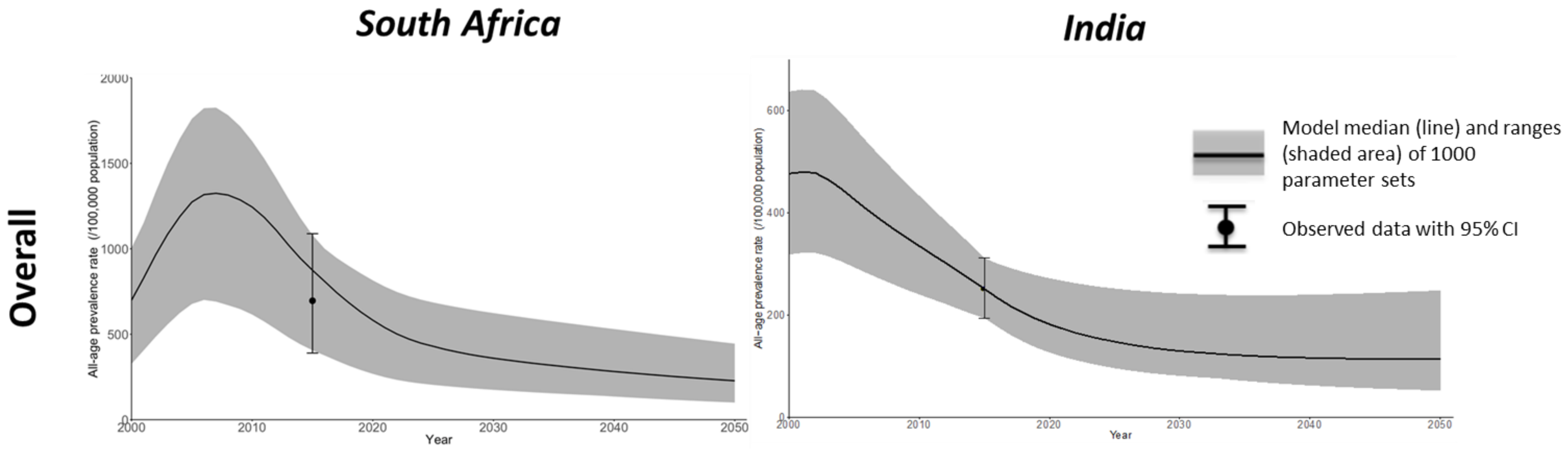

Supplementary Figure 4: TB prevalence projection from calibrated models. Incidence rates shown over time for South Africa and India. Median (line) and ranges (shaded area) summarise 1000 calibrated parameter sets for each country. NB.. Axes differ to aid visualisation.

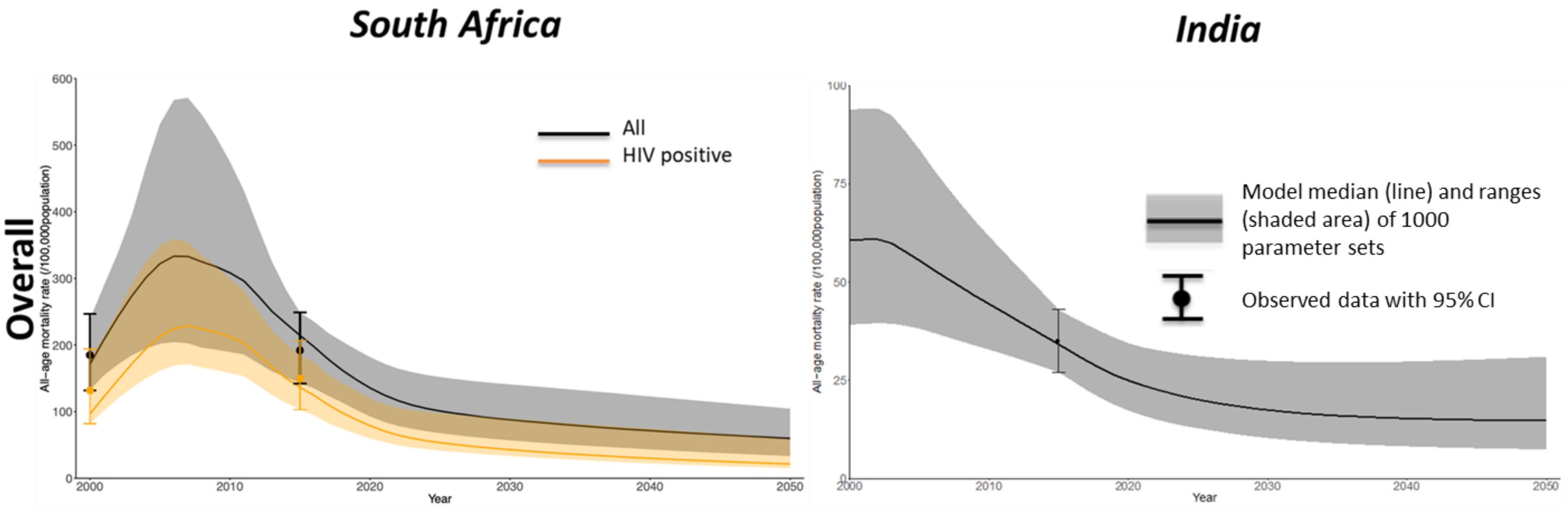

Supplementary Figure 5: Projected all- age mortality rate. Incidence rates shown over time for South Africa and India. Median (line) and ranges (shaded area) summarise 1000 calibrated parameter sets for each country. NB.. Axes differ to aid visualisation.

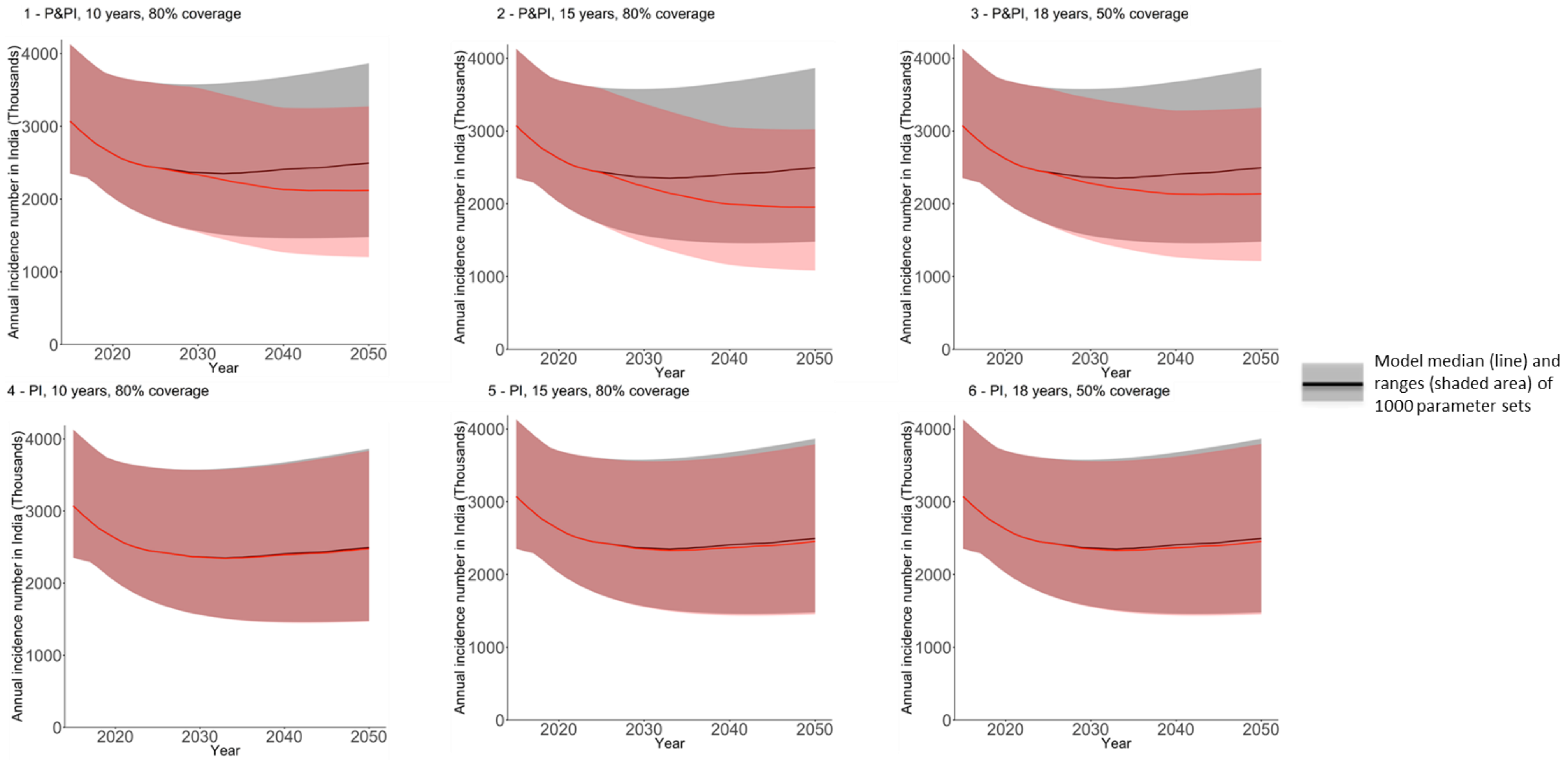

Supplementary Figure 6: Projected change in TB incidence under different vaccination scenarios in India. The top row (panels 1-3) shows scenarios of vaccination with a vaccine with pre- and post-infection efficacy. The bottom row (panels 4-6) shows scenarios of vaccination with a vaccine with post-infection efficacy only. Plots show annual incidence of TB from the 1000 fitted model runs, where the solid black and red lines show baseline and vaccination scenarios, respectively. The bounds of the shaded areas are the upper and lower values of the 1000 runs.

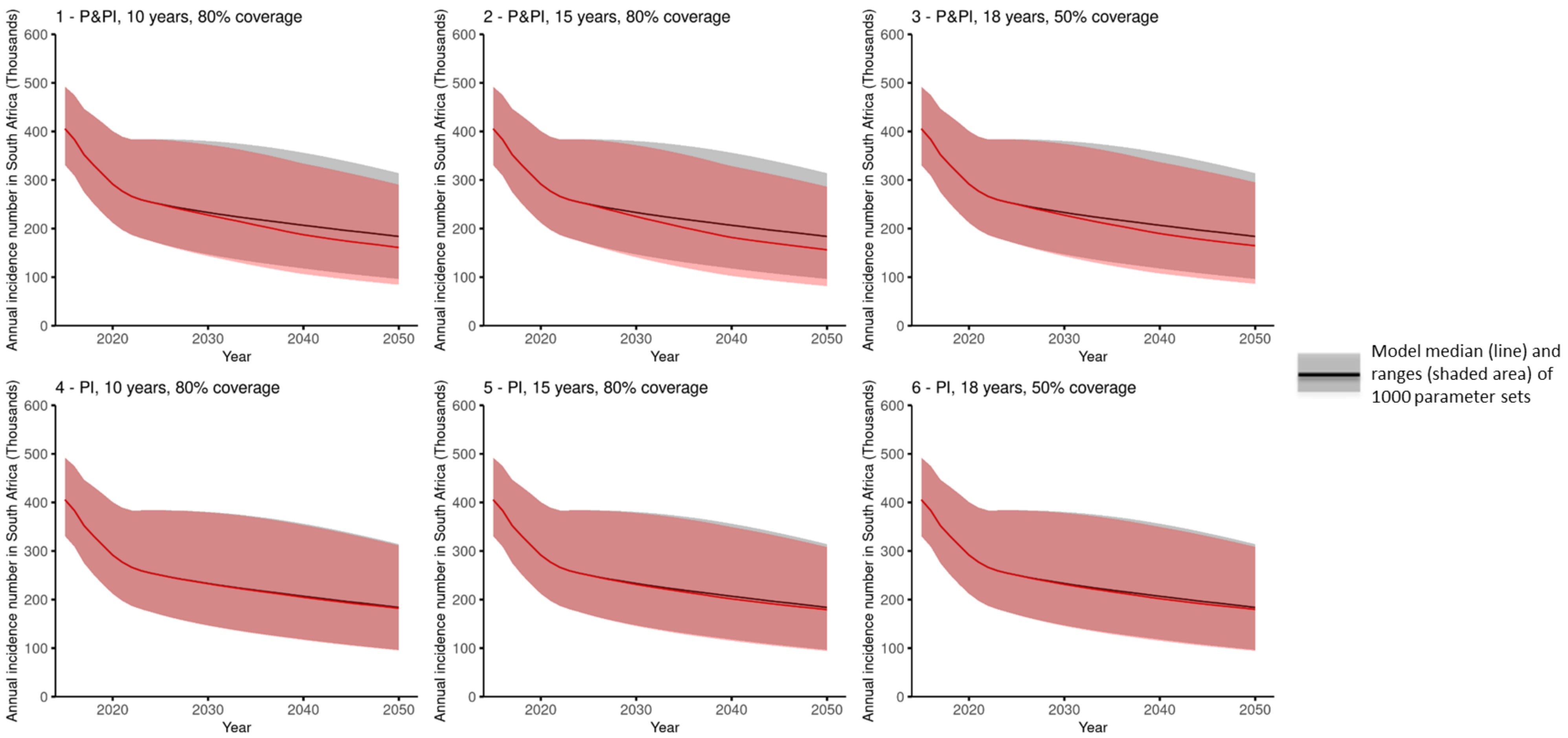

Supplementary Figure 7: Projected change in TB incidence under different vaccination scenarios in South Africa. The top row (panels 1-3) shows scenarios of vaccination with a vaccine with pre- and post-infection efficacy. The bottom row (panels 4-6) shows scenarios of vaccination with a vaccine with post-infection efficacy only. Plots show annual incidence of TB from the 1000 fitted model runs, where the solid black and red lines show baseline and vaccination scenarios, respectively. The bounds of the shaded areas are the upper and lower values of the 1000 runs.

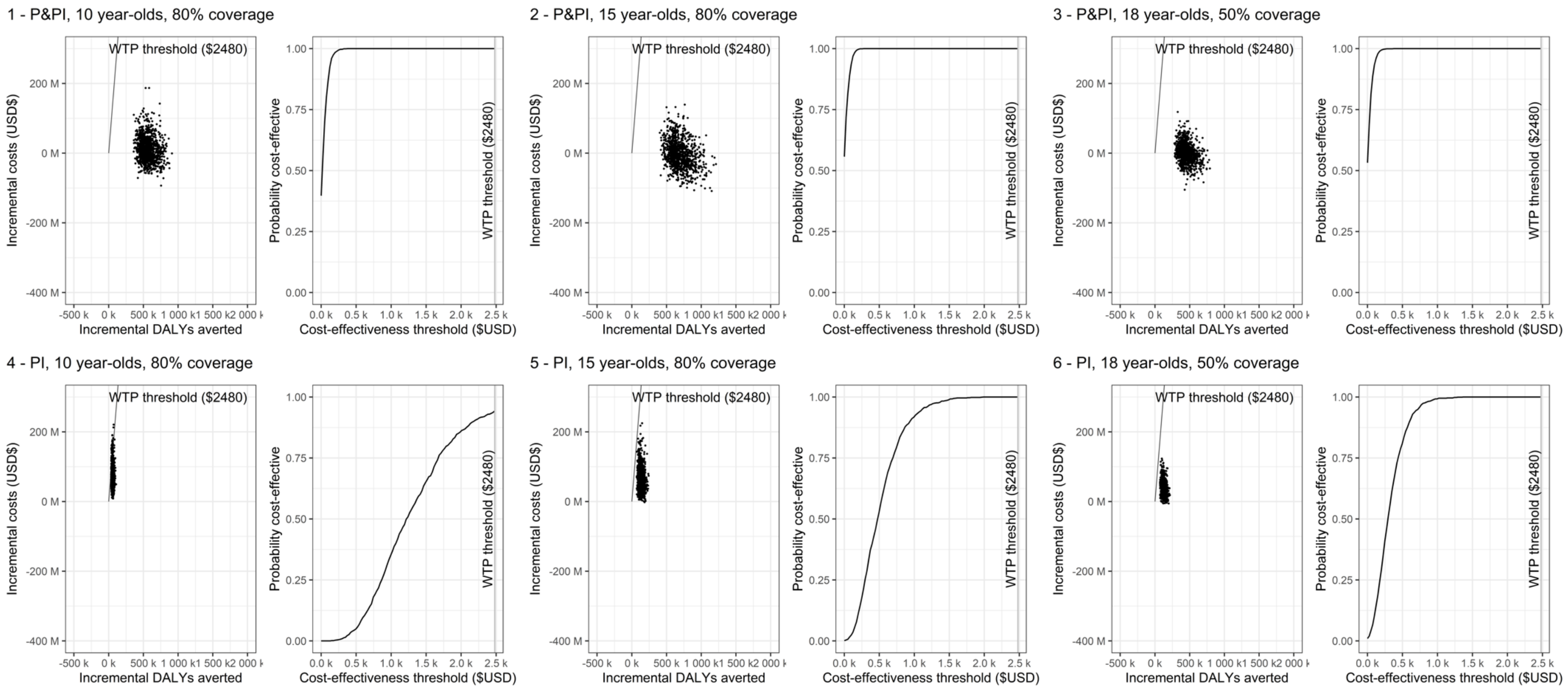

Supplementary Figure 8: Cost-effectiveness planes and cost-effectiveness acceptability curves for vaccines scenarios in South Africa taking a societal perspective. Top row (panels 1-3) shows cost-effectiveness planes (scatter plot) and cost-effectiveness acceptability curves (line graph) for three scenarios with pre- and post-infection vaccine efficacy. Bottom row (panels 4-6) shows cost-effectiveness planes and cost-effectiveness acceptability curves for three scenarios of post-infection -only vaccine efficacy. P&PI denotes vaccine with pre- and post-infection. PI denotes vaccine with post-infection efficacy only. DALY=Disability adjusted life year; USD=United States Dollars, WTP: Willingness to pay.

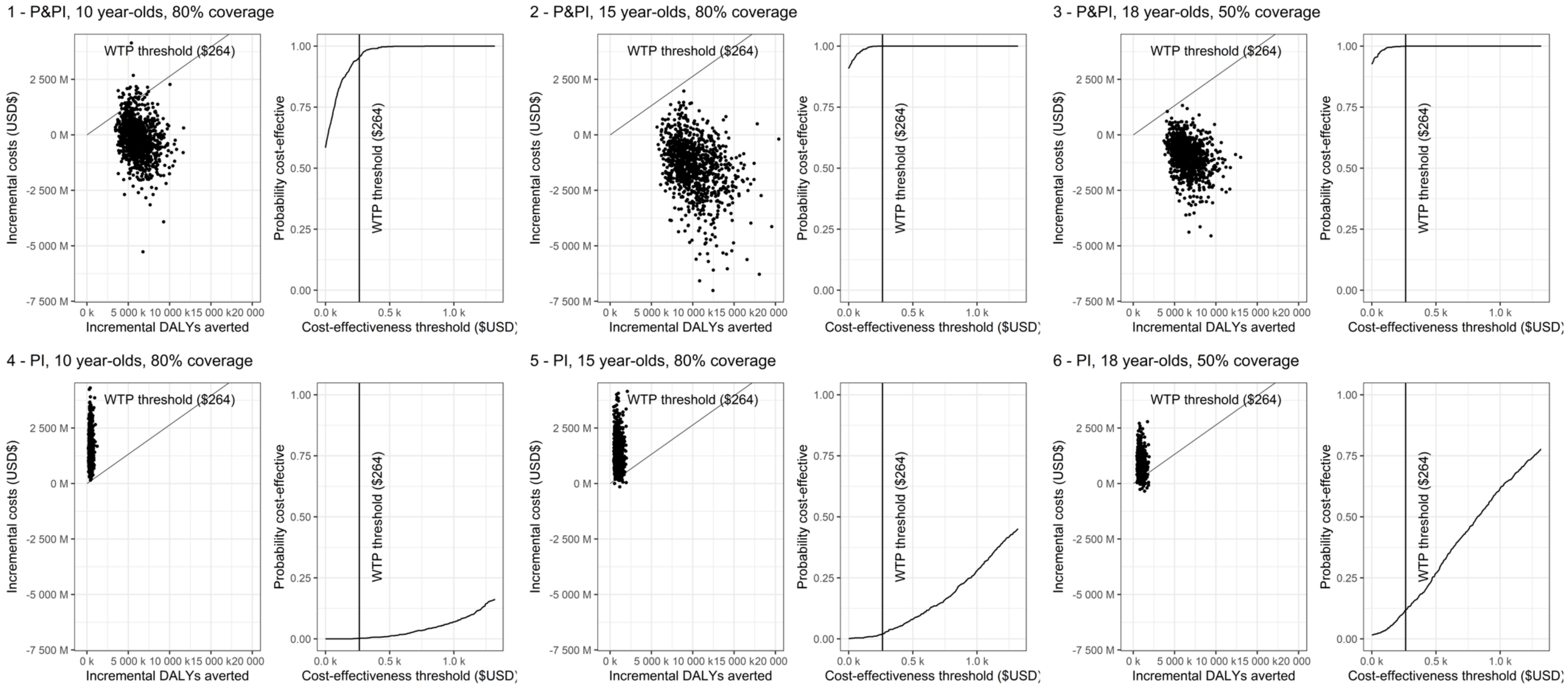

Supplementary Figure 9: Cost-effectiveness planes and cost-effectiveness acceptability curves for vaccines scenarios in India taking a societal perspective. Top row (panels 1-3) shows cost-effectiveness planes (scatter plot) and cost-effectiveness acceptability curves (line graph) for three scenarios with pre- and post-infection vaccine efficacy. Bottom row (panels 4-6) shows cost-effectiveness planes and cost-effectiveness acceptability curves for three scenarios of post-infection -only vaccine efficacy. P&PI denotes vaccine with pre- and post-infection. PI denotes vaccine with post-infection efficacy only. DALY=Disability adjusted life year; USD=United States Dollars, WTP: Willingness to pay.

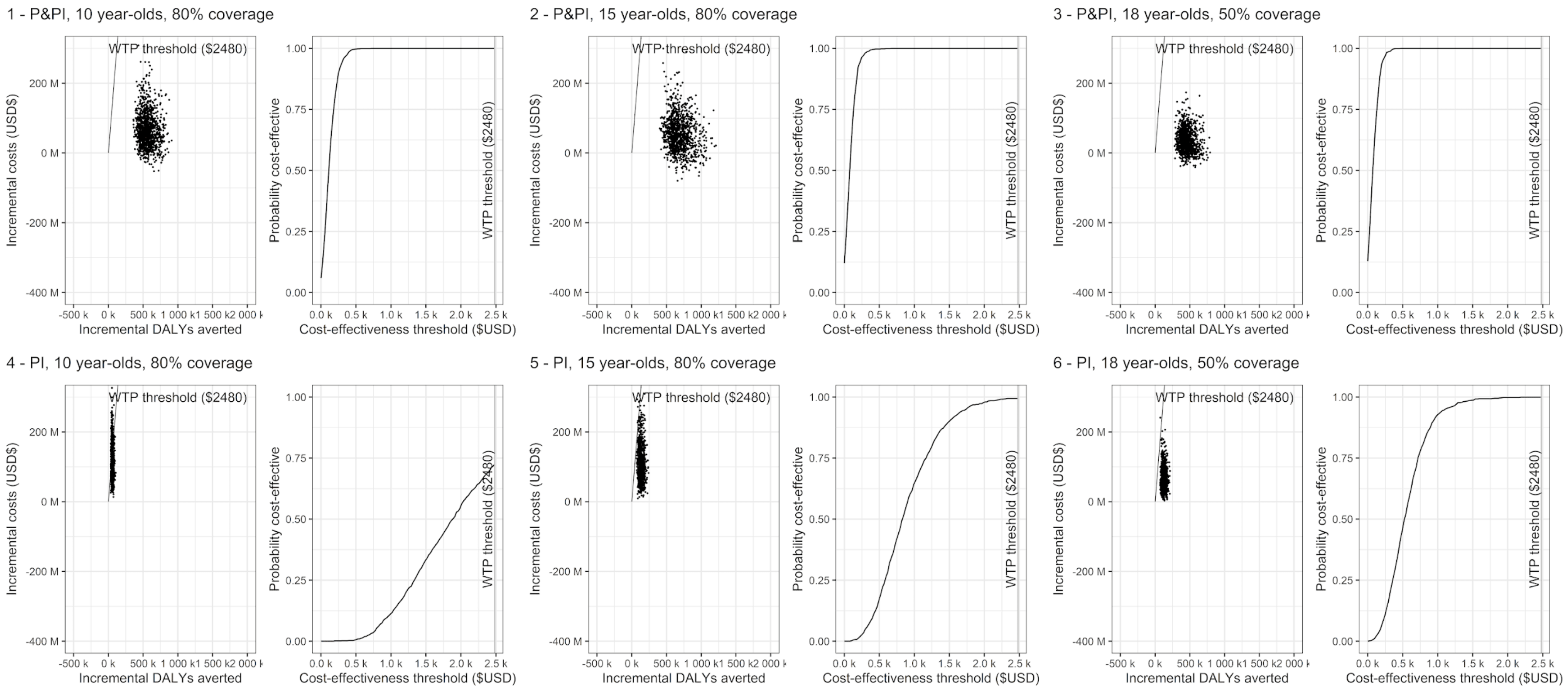

Supplementary Figure 10: Cost-effectiveness planes and cost-effectiveness acceptability curves for vaccines scenarios in South Africa taking a health system perspective, assuming an upper-bound vaccination cost. Top row (panels 1-3) shows cost-effectiveness planes (scatter plot) and cost-effectiveness acceptability curves (line graph) for three scenarios with pre- and post-infection vaccine efficacy. Bottom row (panels 4-6) shows cost-effectiveness planes and cost-effectiveness acceptability curves for three scenarios of post-infection -only vaccine efficacy. P&PI denotes vaccine with pre- and post-infection. PI denotes vaccine with post-infection efficacy only. DALY=Disability adjusted life year; USD=United States Dollars, WTP: Willingness to pay.

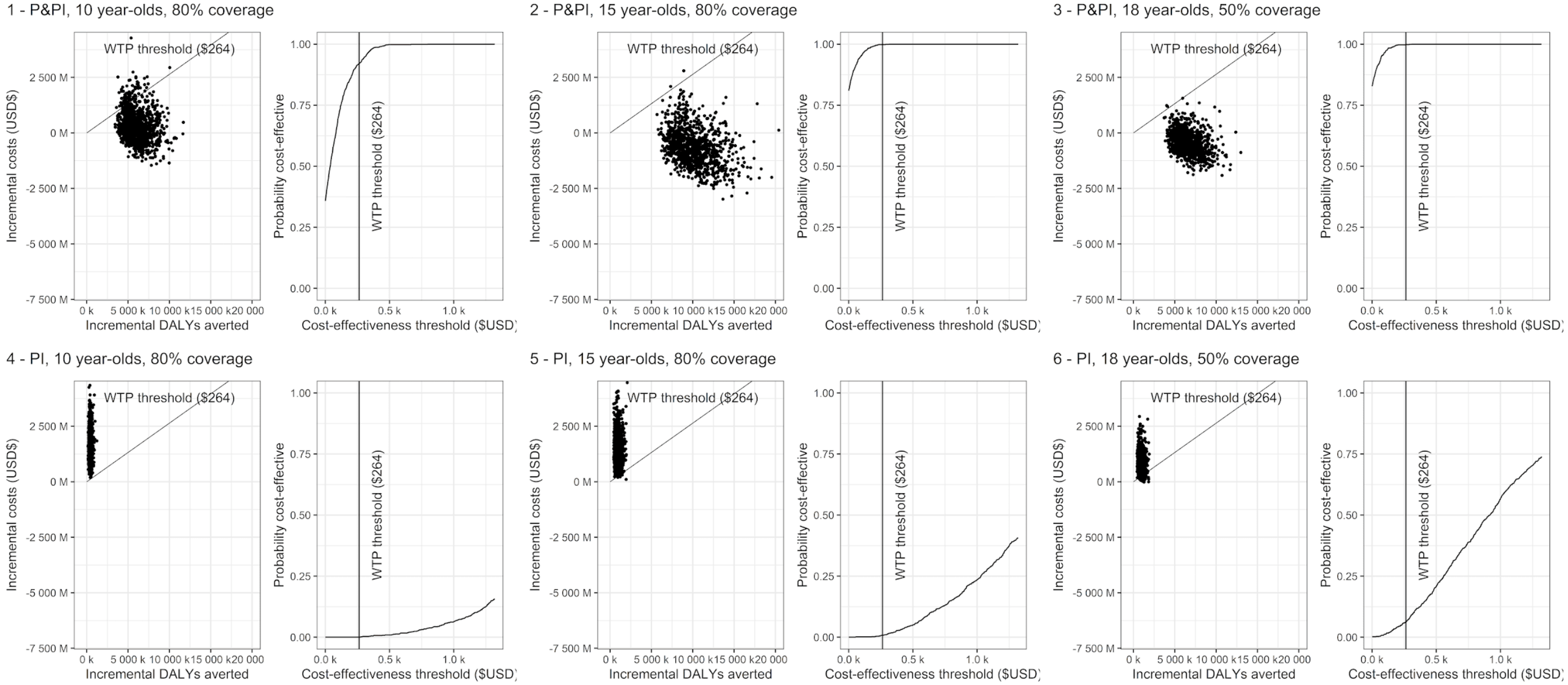

Supplementary Figure 11: Cost-effectiveness planes and cost-effectiveness acceptability curves for vaccines scenarios in India taking a health system perspective, assuming an upper-bound vaccination cost. Top row (panels 1-3) shows cost-effectiveness planes (scatter plot) and cost-effectiveness acceptability curves (line graph) for three scenarios with pre- and post-infection vaccine efficacy. Bottom row (panels 4-6) shows cost-effectiveness planes and cost-effectiveness acceptability curves for three scenarios of post-infection -only vaccine efficacy. P&PI denotes vaccine with pre- and post-infection. PI denotes vaccine with post-infection efficacy only. DALY=Disability adjusted life year; USD=United States Dollars, WTP: Willingness to pay.

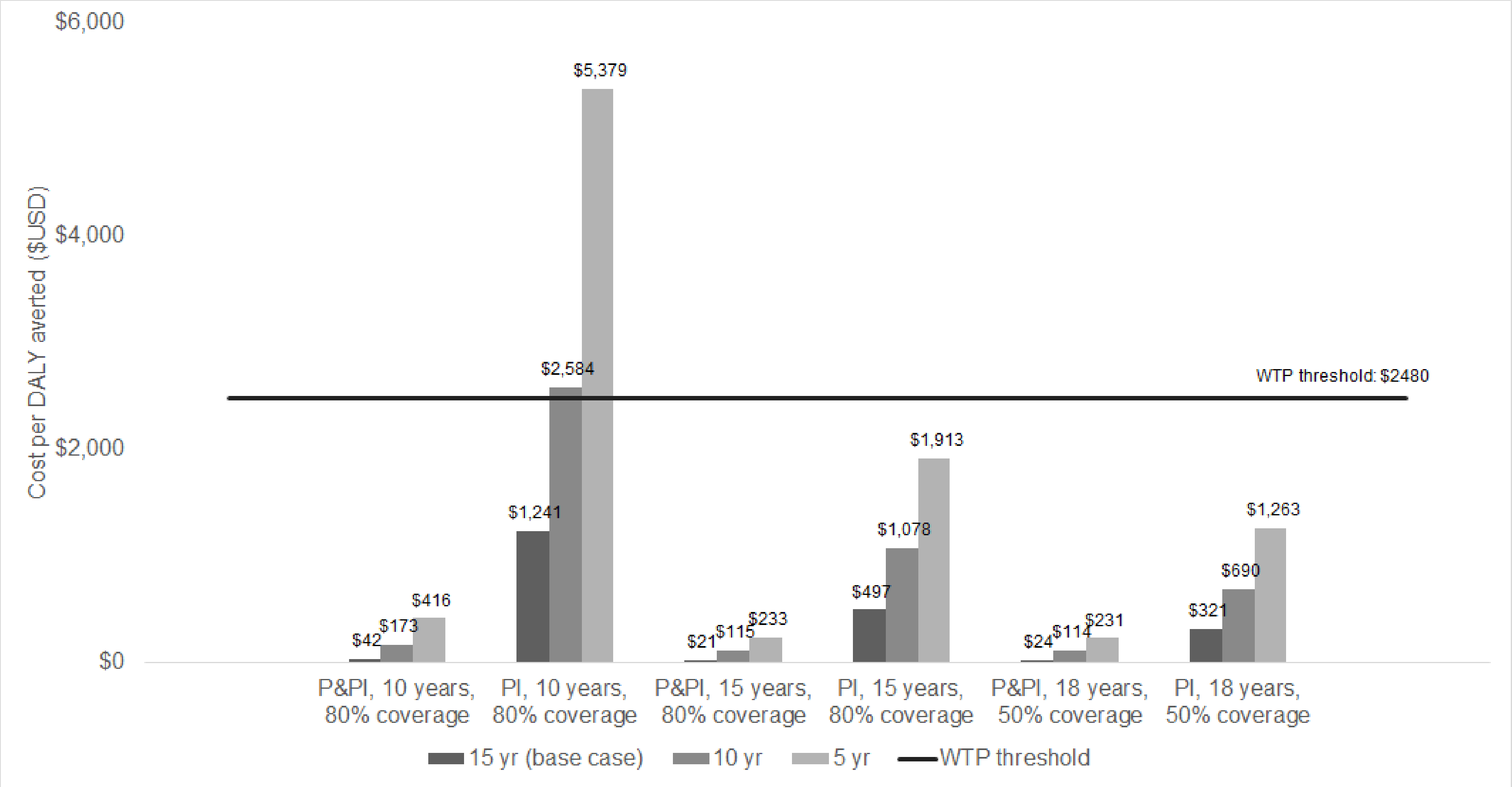

Supplementary Figure 12: Incremental cost-effectiveness ratios for vaccination scenarios in South Africa from a health service perspective, considering vaccines with 5, 10 and 15 year duration of protection. DALY=Disability-adjusted life year; USD=United States Dollars, WTP: Willingness to pay.

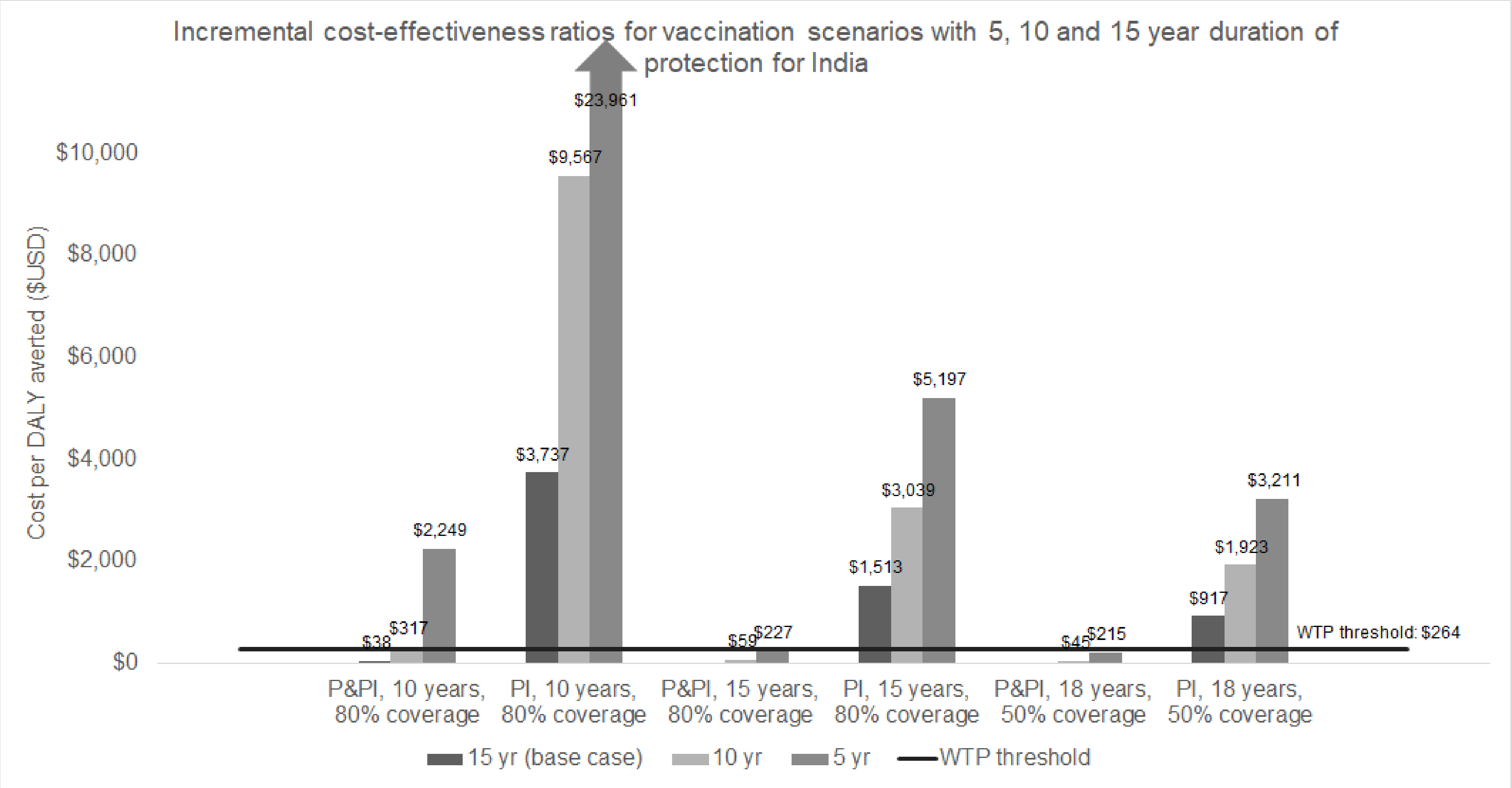

Supplementary Figure 13: Incremental cost-effectiveness ratios for vaccination scenarios in India from a health service perspective, considering vaccines with 5, 10 and 15 year duration of protection

Supplementary Reference list

1. Harris RC, Sumner T, Knight GM, Zhang H, White RG. Potential impact of tuberculosis vaccines in China, South Africa, and India. Science Translational Medicine. 2020;12(564).
2. Vos T, Lim SS, Abbafati C, Abbas KM, Abbasi M, Abbasifard M, et al. Global burden of 369 diseases and injuries in 204 countries and territories, 1990–2019: a systematic analysis for the Global Burden of Disease Study 2019. The Lancet. 2020;396(10258):1204-22.
3. United Nations Development Programme. Human Development Report: Life Expectancy by Country 2020 [Available from: http://hdr.undp.org/en/data.
4. Weerasuriya C, Harris R, McQuaid C, Bozzani F, Ruan Y, Li R, et al. The epidemiologic impact and cost-effectiveness of new tuberculosis vaccines on multidrug resistant tuberculosis in China and India. Submitted. 2020.
5. Bozzani FM, Mudzengi D, Sumner T, Gomez GB, Hippner P, Cardenas V, et al. Empirical estimation of resource constraints for use in model-based economic evaluation: an example of TB services in South Africa. Cost effectiveness and resource allocation. 2018;16(1):1-10.
6. Gotham D, Fortunak J, Pozniak A, Khoo S, Cooke G, Nytko III FE, et al. Estimated generic prices for novel treatments for drug-resistant tuberculosis. Journal of Antimicrobial Chemotherapy. 2017;72(4):1243-52.
7. Siapka M, Vassall A, Cunnama L, Pineda C, Cerecero D, Sweeney S, et al. Cost of tuberculosis treatment in low-and middle-income countries: systematic review and meta-regression. The international journal of tuberculosis and lung disease: the official journal of the International Union against Tuberculosis and Lung Disease. 2020;24(8):802-10.
8. Department of Community Medicine & School of Public Health PGIoMEaRPC, Department for Health Research MoHFW, Health Technology Assessment in India PHFoI, Indian Institute of Techology,Madras, , Tata Institute of Social Science. National Health System Cost Database for India 2020 [Available from: https://www.healtheconomics.pgisph.in/costing_web/index.php?action=Cost_data.
9. Stop TB Partnership. Global Drug Facility Product Catalog 2020 [Available from: http://www.stoptb.org/gdf/drugsupply/product_catalog.asp.
10. Meyer-Rath G, van Rensburg C, Chiu C, Leuner R, Jamieson L, Cohen S. The per-patient costs of HIV services in South Africa: Systematic review and application in the South African HIV Investment Case. PloS one. 2019;14(2):e0210497.
11. Foster N. Structure and agency in the economics of public policy for TB control. Thesis: University of Cape Town; 2019.
12. Chandra A, Kumar R, Kant S, Parthasarathy R, Krishnan A. Direct and indirect patient costs of tuberculosis care in India. Tropical Medicine & International Health. 2020;25(7):803-12.
13. Portnoy A, Vaughan K, Clarke-Deelder E, Suharlim C, Resch SC, Brenzel L, et al. Producing standardized country-level immunization delivery unit cost estimates. PharmacoEconomics. 2020;38(9):995-1005.
14. Moodley I, Tathiah N, Sartorius B. The costs of delivering human papillomavirus vaccination to Grade 4 learners in KwaZulu-Natal, South Africa. South African Medical Journal. 2016;106(5):497-501.
15. Levin A, Wang SA, Levin C, Tsu V, Hutubessy R. Costs of introducing and delivering HPV vaccines in low and lower middle income countries: inputs for GAVI policy on introduction grant support to countries. PloS one. 2014;9(6):e101114.
16. Ministry of Health and Family Welfare GoI. National Strategic Plan for Tuberculosis Elimination 2017-25 New Delhi: Government of India; 2017 [Available from: https://tbcindia.gov.in/WriteReadData/NSP%20Draft%2020.02.2017%201.pdf.

17. Vassall A, Siapka M, Foster N, Cunnama L, Ramma L, Fielding K, et al. Cost-effectiveness of Xpert MTB/RIF for tuberculosis diagnosis in South Africa: a real-world cost analysis and economic evaluation. The Lancet Global Health. 2017;5(7):e710-e9.
18. Rupert S, Vassall A, Raizada N, Khaparde S, Boehme C, Salhotra V, et al. Bottom-up or top-down: unit cost estimation of tuberculosis diagnostic tests in India. The International Journal of Tuberculosis and Lung Disease. 2017;21(4):375-80.
19. Cameron D, Ubels J, Norström F. On what basis are medical cost-effectiveness thresholds set? Clashing opinions and an absence of data: a systematic review. Global health action. 2018;11(1):1447828.
20. Organization WH. Report of the Commission on Macroeconomics and health. 2002.
21. Chi Y-L, Blecher M, Chalkidou K, Culyer A, Claxton K, Edoka I, et al. What next after GDP-based cost-effectiveness thresholds? Gates Open Research. 2020;4(176):176.
22. Ochalek J, Lomas J, Claxton K. Estimating health opportunity costs in low-income and middle-income countries: a novel approach and evidence from cross-country data. BMJ Global Health. 2018;3(6).
